# Dental Practice during COVID-19 in Nepal: A Descriptive Cross-sectional Study

**DOI:** 10.31729/jnma.5022

**Published:** 2020-10-31

**Authors:** Manoj Humagain, Rashmi Humagain, Dinesh Rokaya

**Affiliations:** 1 Department of Periodontology and Oral Implantology, Kathmandu University School of Medical Sciences, Dhulikhel, Nepal; 2 Dental Doctor Clinic, Kathmandu, Nepal; 3 Department of Clinical Dentistry, Walailak University International College of Dentistry, Walailak University, Bangkok, Thailand

**Keywords:** *coronavirus*, *COVID-19*, *dentistry*, *dentists*, *knowledge*

## Abstract

**Introduction:**

Currently, coronavirus disease (COVID-19) has become pandemic and spread globally. In Nepal, the number of COVID-19 is increasing day-by-day. This research was done to find out the impact of COVID-19 on dentists, patients, and dental practice in Nepal.

**Methods:**

This study is a cross-sectional study conducted using an online survey from May 10 to17, 2020. A questionnaire was designed and uploaded in Freeonlinesurveys.com. Following ethical approval, the questionnaire was distributed among 500 dentists, and 406 dentists participated in the study. The survey link was dispersed to the Nepali dentists through social media and e-mail, and the results of the responses were received online. The questionnaire consisted of a total of 34 closed-ended questions containing three parts; demographic details, knowledge of dentists on COVID-19, and the impact of COVID-19 on dentists, patients, and dental treatments.

**Results:**

It showed that majority of the participants were females 243 (60%) of the age group 25-29 years with the clinic as the workplace. Patients receive dental treatments only from 40 (10%) of the dentist. A high number of dentists: 284 (70%) were severely affected by the financial burden and were not receiving a salary during this lockdown. About 349(86%) of the dentist think they should do regular dental treatments, but only 101 (25%) think the dentist should do only dental emergency treatments for COVID-19 infected cases.

**Conclusions:**

Dentists, patients, and dental practice are severely affected by the COVID-19. The majority of the dentists have faced financial burdens. The dental treatments should be done with high standards of care and infection control following proper recommendations.

## INTRODUCTION

A coronaviruses a form of severe acute respiratory syndrome coronavirus 2 (SARS-CoV-2) virus.^1,2^ The virus can transmit via contact with infected people through droplets infection from cough, sneeze, or saliva.^3^ Currently, coronavirus disease (COVID-19) has become pandemic and spread globally.^4,5^ In Nepal, the COVID-19 cases are being increased.^6^ The outbreak of COVID-19 has influenced every aspect of life worldwide and causing a shutdown and self-quarantine.^7^ Vaccines are being tested as a potential therapy, but there are no specific vaccines or other treatments for COVID-19 until now.^8^

The COVID-19 has an impact on dentists, patients, and dental practice. The dentists are at risk of COVID-19 infection due to exposure to hazards such as pathogen exposure, including long working hours, psychological distress, stigma, and fatigue.^9^

This study aimed to find out the impact of COVID-19 in dentists, patients, and dental practice in Nepal.

## METHODS

This study is a cross-sectional study conducted using an online survey from May 10 to 17, 2020. A well-designed questionnaire was designed and uploaded in Freeonlinesurveys.com. The Institutional Review Committee approved the study protocol of the Kathmandu University School of Medical Sciences (28/20). The study was done in dentists in Nepal. The inclusion criteria consist of both male and female Nepali dentists currently residing in Nepal. The survey link was dispersedto the dentists through social media (Facebook, Viber, and Whatsapp) and e-mail, and the results of the responses were received online. Convenient sampling was done. The sample size was calculated as,

$$\begin{array}{ll} \text{n} & \text{= }{\text{Z}}^{\text{2}}\times \text{p}\times \text{(1}-\text{p)}/{\text{e}}^{\text{2}}\\ & \text{= }{\left(1.96\right)}^{\text{2}}\times (0.5)\times (1-0.05)/{\left(0.05\right)}^{\text{2}}\\ & \text{= }384 \end{array}$$

Where,

n = minimum required sample size
Z = 1.96 at 95% confidence interval
p = population proportion, 50%
e = margin of error, 5%

**Figure 1:**
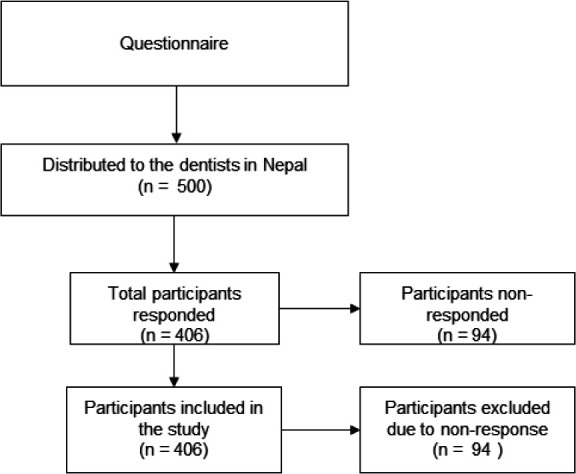
Participation selection overview.

The questionnaire was distributed among 500 dentists, and 406 dentists participated in the study (Figure 1).

The questionnaire consisted of a total of 34 closed-ended questions containing three parts. The first part consists of demographic details, the second part focused on the knowledge of dentists on COVID-19, and the third part gathered information on the impact of COVID-19 in dentists, patients, and dental treatments.

Descriptive statistics were calculated using Microsoft Excel 360 and Statistical Package of the Social Sciences version 20 (IBM Company, Chicago, USA), and the results were expressed as mean and standard deviation.

## RESULTS

The majority of the participants were females, i.e. 244 (60%) of the age group 25-29 years with the clinic as a workplace. Also, the majority of the participants were from Kathmandu, followed by Pokhara, Chabahil, Lalitpur, Janakpur, Jhapa, and Palpa (Table 1).

**Table 1:** Demographic details of the participants.

Demographic details	n (%)
**Gender**	
Males	162 (40)
Females	244 (60)
**Age (years)**	
25-29	230 (57)
30-34	88 (22)
35-39	47 (12)
40-44	26 (6)
45-49	7 (2)
50-54	5 (1)
55-59	2 (0)
>60	1 (0)
**Workplace**	
University	33 (8)
Medical/dental college	126 (31)
Hospital	73 (18)
Private clinic	162 (40)
Other places	12 (3)

The majority of the participants were aware of the COVID-19 and following daily news on COVID-19, and they take necessary precautions of COVID-19. Among them, 345 (85%) were aware of the current CDC or WHO Guidelines for Cross-Infection Control regarding COVID-19 (Table 2).

**Table 2:** Knowledge of COVID-19 in dentists in Nepal.

Questions	Yes n (%)	No n (%)	Don’t know n (%)	Sometimes n (%)
Are you aware of the mode of transmission of COVID-19?	401 (99)	4 (1)	1 (0)	-
Do you think the surgical mask is enough to prevent COVID-19 infection?	37 (9)	337 (83)	32 (8)	-
Have you ever worn an N-90/N-95 mask?	195 (48)	211 (52)	0 (0)	-
Are you updated with the current CDC or WHO Guidelines for Cross-Infection Control regarding COVID-19?	345 (85)	41 (10)	20 (5)	-
Are you following daily news on COVID-19?	370 (91)	4 (1)	-	32 (8)
Are you following the daily cases of COVID-19 (total cases, new, deaths)?	337 (83)	12 (3)	-	57 (14)
Are you taking the necessary precautions of COVID-19 at home?	385 (95)	1 (0)	-	20 (5)
Are you wearing a mask every time when going outside the home?	384 (94)	6 (2)	-	16 (4)
When your friends or relatives visit your home during the lockdown, do they wear a mask every time?	252 (62)	57 (14)	-	97 (24)

Among the participants, 97 (24%) of the dentists were not going to the workplace during the lockdown. Patients receive dental treatments only from 10% of the dentist in lockdown. It also showed that a high number of the dentist 284 (70%) were severely affected by the financial burden and were not receiving a salary during this lockdown as most dentists (over >90%) work in the private sector. About 350 (86%) of the dentist think they should do regular dental treatments, but only 102 (25%) think the dentist should do dental emergency treatments for the COVID-19 infected cases. About 187 (46%) of the dentists use personal protective equipment (PPE) and hand hygiene practices for every patient. It showed that 215 (53%) dentists are you involved in online teaching and learning activities during the lockdown (Table 3).

**Table 3:** Impact of COVID-19 on the dentists, patients, and dental treatments in Nepal.

Questions	Yes n (%)	No n (%)	Don’t know n (%)	Sometimes n (%)
Are you going to the workplace during the lockdown?	97 (24)	235 (58)	-	73 (18)
Are you doing any dental treatment in lockdown?	40 (10)	305 (75)	-	61 (15)
Are you facing financial burdens because of the lockdown?	284 (70)	102 (25)	-	20 (5)
Are you receiving a salary during this lockdown?	105 (26)	248 (61)	53 (13)	-
Are you deferring regular dental treatment of patients showing suspicious symptoms?	304 (75)	65 (16)	37 (9)	-
Are you deferring emergency dental treatment of patients showing suspicious symptoms?	167 (41)	146 (36)	93 (23)	-
Are you aware of the necessary precautions of COVID-19 in the clinic?	353 (87)	20 (5)	33 (8)	-
Are you taking the necessary precautions of COVID-19 in the clinic?	227 (56)	85 (21)	93 (23)	-
Are you currently asking every patient’s travel history before performing dental treatment?	350 (86)	24 (6)	37 (9)	-
Do you think the dentist should do regular patients in the pandemic period?	28 (7)	350 (86)	28 (7)	-
Do you think the dentist should do dental emergency treatments in COVID-19 infected cases?	240 (59)	101 (25)	65 (16)	-
Do you ask every patient to rinse the mouth with a mouthwash (0.2% povidone-iodine or 0.5-1% hydrogen peroxide mouth rinse) before treatment?	268 (66)	118 (29)	20 (5)	-
Do you wash hands with soap and water/use sanitizer before and after the treatment of every patient?	398 (98)	8 (2)	0 (0)	-
Do you use rubber dam isolation for every patient for COVID-19 prevention?	24 (6)	337 (83)	45 (11)	-
Do you use personal protective equipment (PPE) and hand hygiene practices for every patient?	207 (51)	187 (46)	12 (3)	-
Have you ever worn an N-90/ N-95 mask while treating a patient in your dental practice?	122 (30)	272 (67)	12 (3)	-
Do you think the N-90/ N-95 mask should be routinely worn in dental practice due to the current outbreak?	366 (90)	28 (7)	12 (3)	-
Are you currently taking every patient’s body temperature before performing dental treatment?	207 (51)	150 (37)	-	49 (12)
Are providing information to the patient’s regarding COVID-19?	337 (83)	24 (6)	-	45 (11)
Are you involved in online teaching and learning activities during a lockdown?	215 (53)	114 (28)	-	77 (19)

**Figure 2:**
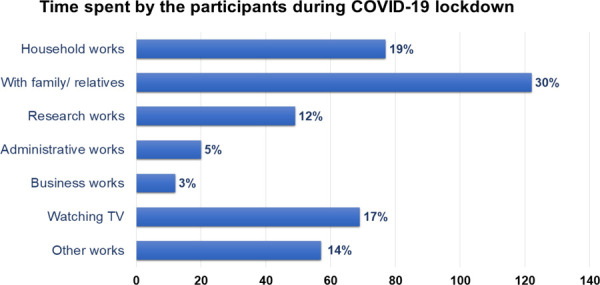
Time spent by the dentists during the lockdown in Nepal.

The majority of the dentists (>30%) spend time with family and relatives followed by household works 77 (19%), watching TV 69 (17%), research works 49 (12%), administrative works 20 (5%), business work 12 (3%), and other works 57 (14%) (Figure 2).

## DISCUSSION

Pandemic disease and disasters have an impact on a country's economy, social life, education, general health, oral health, etc.^10–12^ Currently, each country has been rapidly developing policy to manage the COVID-19 epidemic taking reference from the WHO but interpreting in different ways. There are various public health challenges in controlling the spread of COVID-19 in Nepal.^13^ In Nepal, the cases are being increased daily. Moreover, there is a shortage of testing kits, medical supplies, personal protective equipment, and poor reporting are major challenges to be tackled in the case of the COVID-19. Additionally, some COVID-19 cases remain asymptomatic, so it is difficult to predict the severity of the outbreak. This study done in Nepal showed that dentists and patients are severely affected. A high number of the dentist (70%) are faced by financial burden and were not receiving a salary during this lockdown as most dentists (over >90%) work in the private sector. Also, dentists are at risk of COVID-19 infection. These factors result in psychological distress and stigma.^9^

The UK National Health Service's (NHS's) mentioned that the dentists should continue to provide routine care for asymptomatic patients with no close contact history.^14^ Meanwhile, many dentists felt uncomfortable with the NHS's advice and postponed the routine care for spreading the COVID-19 among their patients. However, in the US, the multiagency bureaucratic system has been blamed for its slow response. Still, the many dentists have postponed the regular dental treatments. There can be various dental emergency cases such as orofacial trauma causing avulsion, severe pulpitis, TMD pain, periodontal cases, etc.^15–18^ Hence, dentists show be able to handle the dental emergency cases although they can postpone regular dental treatments. In this study, it showed that only 10% of the dentist are doing dental treatments especially emergency and urgent cases in lockdown. Hence, only a few dentists are available to provide dental treatment to the patient. These also showed that the dental treatments of the patients are also being affected by the COVID-19 infection.

In dental settings, there is a high risk of crossinfection between patients and dental practitioners, and effective infection control protocols are urgently needed.^16^ In this present study, the dentists in Nepal have good knowledge ofCOVID-19. Over 85% of the Nepali Dentists were aware of the current CDC or WHO Guidelines. Use of personal protective equipment, hand hygiene practices, mouth rinsing, disposable instruments, and use of rubber dam, reducing ultrasonic instruments use. The treatment component should be strengthened to reduce the transmission and case fatality. Good knowledge and positive attitude of health care staff towards COVID-19 and can help dentists in providing safe dental treatment.

## CONCLUSIONS

Dentists, patients, and dental practice are severely affected by the COVID-19. Over 70% of the dentist have faced financial burden. Most of the dentists had good knowledge of COVID-19 and following daily news. The dental treatments should be done with high standards of care and infection control following proper recommendations.

## Conflict of Interest

**None.**
